# Association of American Medical Editors

**Published:** 1874-06-15

**Authors:** 


ASSOCIATION OF AMERICAN MEDICAL EDITORS.—This Association met in the office of Dr. W. Brodie, Detroit, June 1, 1874, at 7½ P. M. The meeting was called to order by Dr. N. S. Davis, and the President, Dr. W. K. Bowling, of New York, was conducted to the chair. The minutes of the last meeting were read by the acting Secretary and approved.

The following members were present: Drs. N. S. Davis, of Chicago; W. T. Briggs and W. K. Bowling, of Nashville; W. S. Edgar, of St. Louis; T. M. Stevens, of Indianapolis; Leartus Connor, of Detroit; W. T. Taylor, of Philadelphia; J. M. Toner, of Washington; and E. Lloyd Howard, of Baltimore. The President, Dr. Bowling, then delivered a very interesting address, a copy of which was requested for publication. Dr. W. S. Edgar read a paper in opposition to the establishment of State Universities for the education of professional men, and especially medical men. He claimed that it was not only unfair to tax the whole people for the education of a few in the higher and more special branches of learning, but that the establishment of medical schools as departments of State universities often caused them to be located in small towns where there can be no facilities for clinical instruction, and no adequate field for a competent corps of teachers to practice in. The paper elicited some remarks by Drs. N. S. Davis and T. M. Stevens, after which the following officers were elected for the ensuing year:

*President*—Dr. W. S. Edgar, of St. Louis.
*Vice President*—Dr. L. Connor, of Detroit.
*Secretary*—Dr. F. H. Davis, of Chicago.

The Association then adjourned to meet on the Monday evening previous to the first Tuesday in May, 1875, in Louisville, Kentucky.

